# Image Analysis for the Quantitative Comparison of Stress Fibers and Focal Adhesions

**DOI:** 10.1371/journal.pone.0107393

**Published:** 2014-09-30

**Authors:** Alberto Elosegui-Artola, Alvaro Jorge-Peñas, Oihana Moreno-Arotzena, Amaia Oregi, Marta Lasa, José Manuel García-Aznar, Elena M. De Juan-Pardo, Rafael Aldabe

**Affiliations:** 1 Tissue Engineering and Biomaterials Unit, Centro de Estudios e Investigaciones Técnicas and Tecnun, University of Navarra, San Sebastian, Spain; 2 Multiscale in Mechanical and Biological Engineering, Aragón Institute of Engineering Research, Universidad de Zaragoza, Zaragoza, Spain; 3 Gene Therapy and Hepatology Area, Center for Applied Medical Research (CIMA), University of Navarra, Pamplona, Spain; Instituto Gulbenkian de Ciência, Portugal

## Abstract

Actin stress fibers (SFs) detect and transmit forces to the extracellular matrix through focal adhesions (FAs), and molecules in this pathway determine cellular behavior. Here, we designed two different computational tools to quantify actin SFs and the distribution of actin cytoskeletal proteins within a normalized cellular morphology. Moreover, a systematic cell response comparison between the control cells and those with impaired actin cytoskeleton polymerization was performed to demonstrate the reliability of the tools. Indeed, a variety of proteins that were present within the string beginning at the focal adhesions (vinculin) up to the actin SFs contraction (non-muscle myosin II (NMMII)) were analyzed. Finally, the software used allows for the quantification of the SFs based on the relative positions of FAs. Therefore, it provides a better insight into the cell mechanics and broadens the knowledge of the nature of SFs.

## Introduction

The tension generated in the actin cytoskeleton determines cellular adhesion, spread area, motility, proliferation, differentiation and apoptosis. Cellular adhesion is a complex process in which transmembrane receptors such as integrins are recruited, activated and consequently bind to the extracellular matrix [1], [2]. The actin cytoskeleton rapidly interacts with integrins because these receptors cluster with FAs, thus anchoring the actomyosin cytoskeleton to the extracellular matrix and forming what are known as SFs. SFs are long and aligned contractile actin bundles that are characterized by repeated units of NMMII and several actin-binding proteins. Through these interactions, FAs and integrins feel and balance cellular-extracellular matrix forces [3]–[6]. NMMII motor proteins slide over the actin filament generating contraction of the SFs [7]–[11], whereas FAs are supramolecular complexes located in the cell periphery and more centrally in less motile regions [5]. Mammalian cells contain at least three different categories of SFs including transverse arcs, dorsal SFs and ventral SFs [12]. Ventral SFs are the only bundles that are bound at both ends to FAs and thus play a major role in cell adhesion and contraction [13].

Indeed, SFs and FAs are interactive structures that are essential for cell shape. Unraveling the linkage between SFs and FAs provoked the development of different approaches. Specifically, image-based analytical tools allow for both systematic and quantitative studies. Those tools have been used to perform high-throughput segmentation and quantification of FAs in several studies [14]–[16]. In terms of SFs, different approaches have been used including measuring the percentage of cells with long long fibers [17], analyzing the local retraction of SFs after photobleaching or nanosurgery [18], or image filtering to enhance SF visibility through intensity thresholds [19] or noise reduction [20]. Additional approaches have been employed to analyze the distribution of these structures across the whole cell body.

For instance, images have been used to demonstrate the locations of F-actin and to study the spacing between other critical proteins such as α-actinin and myosin [21], and several groups have used the sum of phalloidin intensity across the cell as a parameter of the actin structure [22]. However, due to the high degree of heterogeneity in cell morphology, it is difficult to compare different cells and conditions. Recently, a cell shape normalization approach was proposed to analyze the distribution of actin, FA dynamics and traction force with respect to the direction of cell migration [23]. Another recent approach defines one fluorescence intensity line per cell, which spans from the perinuclear region to the cell edge, to study nanoparticle fluorescence emission [24].

Despite these approaches, there is currently no objective, automated algorithm to quantify the amount and distribution of cytoskeletal structures. The main purpose of this work is therefore to present the development of a computational tool for the analysis and quantification of cytoskeletal characteristics. First, a new complementary method to quantify the number of SFs in 3D has been designed that takes advantage of both vinculin- and actin-stained images. To our knowledge, no previous work has directly quantified the number of actin SFs from an unprocessed actin-stained image. Second, we define a new parameter for evaluating intracellular structure distribution, thus allowing for comparisons between cells under different conditions independent of the shape and spread of the cells. We have to keep in mind that both the number and organization of intracellular structures can play a significant role. This tool allows an unbiased comparison of any protein or cellular structure.

This combination of tools is the first to characterize cytoskeletal properties by improving the detection and interpretation of the most important differences among singular biological conditions.

## Materials and Methods

The presented novel computational tools have been applied for the systematic study of the cell structure and quantification of actin SFs. The analytical tools are divided into three main sections, which include the study of cellular morphometric features followed by the explanation of the two novel computational tools. Both the quantification of actin SFs and the study of protein distribution from different angles are explained in detail. Finally, the common experimental methods used are described.

### Computational Tools

Unless otherwise specified herein, the acquired images were processed and analyzed using custom-made code written in Matlab (The Mathworks Inc., Natick, MA, USA) to take advantage of its image visualization and processing capabilities.

#### Morphometric analysis

The spread area and circularity of the cells were measured immediately after fixing and staining with Alexa Fluor 488 phalloidin (Invitrogen). Images were then taken with a Nikon Ti-Eclipse microscope using a 10× objective. NIS Elements (Nikon, Melville, NY, USA) software was used to measure both the spread area and the aspect ratio of the cells. For the measurements of cell traction forces, polyacrylamide hydrogels of 10 kPa were manufactured (Supplementary Materials and Methods in File S2). The corresponding map of cell forces was calculated using a previously described Fourier transform algorithm [25]–[26] (Supplementary Materials and Methods in File S2).

#### Quantification of actin SFs

*Preprocessing and analysis of FAs*: A 3D data set was acquired from an Axiovert 200 M confocal LSM 510 META Zeiss microscope. The images in the data set were first reduced to 2D images by applying a maximum intensity projection along the z-axis to the optical sections. Vinculin-labeled images were subjected to an additional 2-step filtering process, to both suppress the effects of the soluble vinculin in the cytosol and enhance FAs. Specifically, a median filter was applied to reduce the background noise, followed by a top-hat filtering step to enhance the bright spots.

FAs were characterized using the NIS-Elements imaging software (Nikon, Melville, NY, USA). This software was used to compute four variables from the preprocessed and segmented vinculin-labeled images, including the area, circularity, mean intensity and density per cell. Particularly, circularity was calculated as 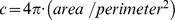, where _0≤*c*≤1_, with *_c_*_ = 0_ for a straight line and *_c_*_ = 1_ for a perfect circle. The size and vinculin content of individual FAs are proportional to the local force applied to these structures by the contractile system of the cell [27]. The circularity of the FAs is related to the stress that the cell is supporting, and the FAs become increasingly less circular when the cells support higher tension [17].

*Actin SFs three dimensional segmentation*: Individual actin SFs were automatically quantified using a novel approach that combines the information derived from preprocessed vinculin and phalloidin images. Considering that actin SFs terminate at discrete FAs, the proposed tool searches for the connecting link between each pair of FAs that best fits the phalloidin image. Because the SFs are under tension, SFs have to be straight filaments without buckling. Nevertheless, in addition to pure straight lines, the method also considers slightly curved links, mainly because some small FAs could have been erased in the previous filtering step. Additionally, the SFs could be bent by binding to other structures, such as other SFs. First, all the possible SFs are detected in the maximum intensity projection of the phalloidin images. Thus, assuming just one connecting link between the n-th pair of FAs *p_n_*(*FA_i_*, *FA_j_*), a second-order polynomial of the form *y* = *a_n_x* ^2^+*b_n_x+c_n_*, was used as a fitting model, where 
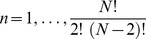
, 1≤*i*<*j*≤*N*, and N is the total number of FAs per segmented cell. This polynomial must pass through the points (*x_i_*,*y_i_*) and (*x_j_*,*y_j_*) located at the centroids of *FA_i_* and *FA_j_*, respectively, and it would be reduced to a straight line for *a_n_* = 0. To find the coefficients (*a_n_*_,_ *b_n_*_,_ *c_n_*), a fitting criteria based on the grey-scale intensity *I*(*x,y*) of the phalloidin-labeled image was used, i.e, 
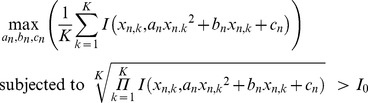
where (*x_n,k_*,*y_n,k_*) is the k-th point in the path defined by the n-th fitting polynomial between *FA_i_* and *FA_j_* and *I*_0_ is a threshold defined by the user according to the amount of background noise in the phalloidin image. In other words, from all possible polynomials whose paths have geometric mean intensity larger than *I*_0_, the one that presents the path with the highest mean intensity is selected.

Then, all the possible SFs are evaluated in the Z-axis. The Z stack for every point of each possible SF is obtained. Each SF results in a two-dimensional image (l,k) where l is the length and k the height for every point. First, the FAs are located. Then, the same previously performed procedure to find the best polynomial fit is followed. The study of the SFs in the Z-axis eliminates all the possible SFs that do not end between the two FAs under study. The expansion to 3D eliminates the 18.57±7.49% of SFs quantified in 2D.

In summary, all the SFs are segmented depending only on three parameters: minimum length, maximum buckling and minimum mean intensity of the SF. Because one-dimensional fitting polynomials were used, the width of the SFs was not considered.

#### Local intensity: quantification of the structures' distribution

To quantify the distribution of cytoskeletal structures we defined three parameters, the Radial Mean Intensity (RMI), Local Ring Mean Intensity (LRMI), and Constant Area Ring Intensity Percentage (CARIP).

*Radial Mean Intensity (RMI):* We defined RMI to quantify protein distribution independently of cell shape and size. To this end, we transformed cell morphology into a circular distribution, removing the influence of shape and size. This circular distribution is obtained by applying to each local intensity pixel a geometric transformation, which depends only on the distance of the pixel to the centroid and the closest edge of the cell. First, The cell image is transformed from Cartesian (x,y) to polar (*r*,*θ*) coordinates centered at the cell centroid C. Then and for all the pixels with a given angle *θ*, a normalized radius *r_p_* is calculated as *r_p_* = r/*R*_max_, where *R*_max_ is the value of r corresponding the cell edge along direction *θ*. Since we considered both migrating and non-migrating cells, the origin of the angular axis θ is arbitrary, although it could be set to a given cell axis if polarized cells were studied specifically. Finally, intensity values for each value of *r_p_* (ranging from 0, the cell centroid, to 1, the cell edge) were averaged for all values of θ to calculate the RMI. Thus, the RMI quantifies the average intensity of a given protein as a function of the relative distance to the cell centroid and edge.

*Local Radial Mean Intensity (LRMI)*: To allow for quantitative statistical comparisons, RMI values for a given cell were averaged in 5 bins of the normalized radius *r_p_* (*r_p_* = 0–0.2,0.2–0.4,…,0.8-1). Roughly, the first section (*r_p_* = 0–0.2) encloses the nucleus and the nuclear membrane. The following two sections (*r_p_* = 0.2–0.6) are composed of various SFs, including transverse arcs, dorsal SFs and ventral SFs. The last two sections (*r_p_* = 0.6–1) are related to the leading and trailing edges, which contain lamellipodia, filopodia and the end of the main ventral SFs.

*Constant Area Ring Intensity Percentage (CARIP)*: Since the different bins defined to establish the LRMI can have different areas, we also established an alternative binning of r_p_. In this case, instead of defining 5 uniform bins from *r_p_* = 0 to *r_p_* = 1, we modified the thresholds in *r_p_* to produce regions which contained the same cell area. The CARIP was then defined as the percentage of total protein intensity localized in each equal-area bin.

### Experimental Samples

#### Cell culture

HeLa cells (epithelial cervix adenocarcinoma; ATCC CCL-2) were at 37°C in 5% CO_2_ cultured in Glutamax-containing DMEM supplemented with 10% (vol/vol) dialyzed FBS (Invitrogen), 100 units/mL penicillin (Invitrogen), and 100 µg/mL streptomycin (Invitrogen). The cells were grown in media containing 80 µM of natural (12C6) or 13C615N4 L-arginine (Cambridge Isotope Labs. The cells were cultured for at least six population doublings to ensure the complete incorporation of the labeled arginine.

#### Immunofluorescence analysis

For the cytoskeletal protein staining, HeLa cells were fixed with 4% paraformaldehyde, permeabilized with 0.1% Triton X-100 and incubated with the primary anti-vinculin antibody (Sigma), an anti-mouse IgG CY3 conjugate developed in sheep (Sigma), an anti-NMMIIA (Abcam) and the Alexa Fluor 488 donkey anti-rabbit IgG (Invitrogen). All of the incubations were performed for 30 min at 37°C. To stain actin SFs, cells were incubated with Oregon Green 448 phalloidin (Invitrogen) for 15 min at 37°C. The stained cells for each preparation were mounted with Vectashield Mounting Medium with DAPI (Vector Laboratories). The samples were analyzed using the 63× objective of an Axiovert 200 M confocal LSM 510 META Zeiss microscope.

#### Statistical analysis

The results are expressed as the mean ± standard deviation (SD). The difference between the two mean values was analyzed using two-tailed Student's t-test, and *P*<0.05 was considered significant. If the normality test failed, the Mann-Whitney U test was performed. The coefficient of determination (R^2^) was used to study the cross correlation between different data sets. The aim of the computational tools is to unravel the role of different critical parameters in the cellular behavior. As an example, we compared not only HeLa control cells but also cells where actin polymerization was inhibited by adding Cytochalasin B (Cyt B, Sigma-Aldrich). It is known that the pharmacological inhibition of cellular contractility disrupts the actin SFs [28]–[29] and FAs [30]. The treatment lasted 96 h, and we used a concentration of 0.6 µM Cyt B diluted in DMSO, which disrupted the actin cytoskeleton. Because the concentration of Cyt B was small, it reduced the toxicity compared to the toxicity used in previous studies [30]–[31]. Thus, the effect on the cytoskeleton was less aggressive with no apoptotic induction. The effect of 0.6 µM Cyt B stabilizes after 72 to 96 h. The computational tools were applied to compare the effect of Cyt B to the DMSO control on the actomyosin cytoskeleton. Thus, all the statistical analysis were performed to compare between Cyt B and DMSO conditions.

## Results

The results are divided into three main sections. First, the cellular morphometric features were measured to identify structural differences. Then, the new software was applied to quantify the SFs. Moreover, correlations between the SFs with FAs and cellular properties were analyzed. Finally, after cellular shape was normalized, the local intensity and distributions of vinculin, actin and NMMII across the cell were calculated. All reported results were obtained with the same set of parameters for image processing and analyzing.

### Morphometric analysis

**Figure 1:**
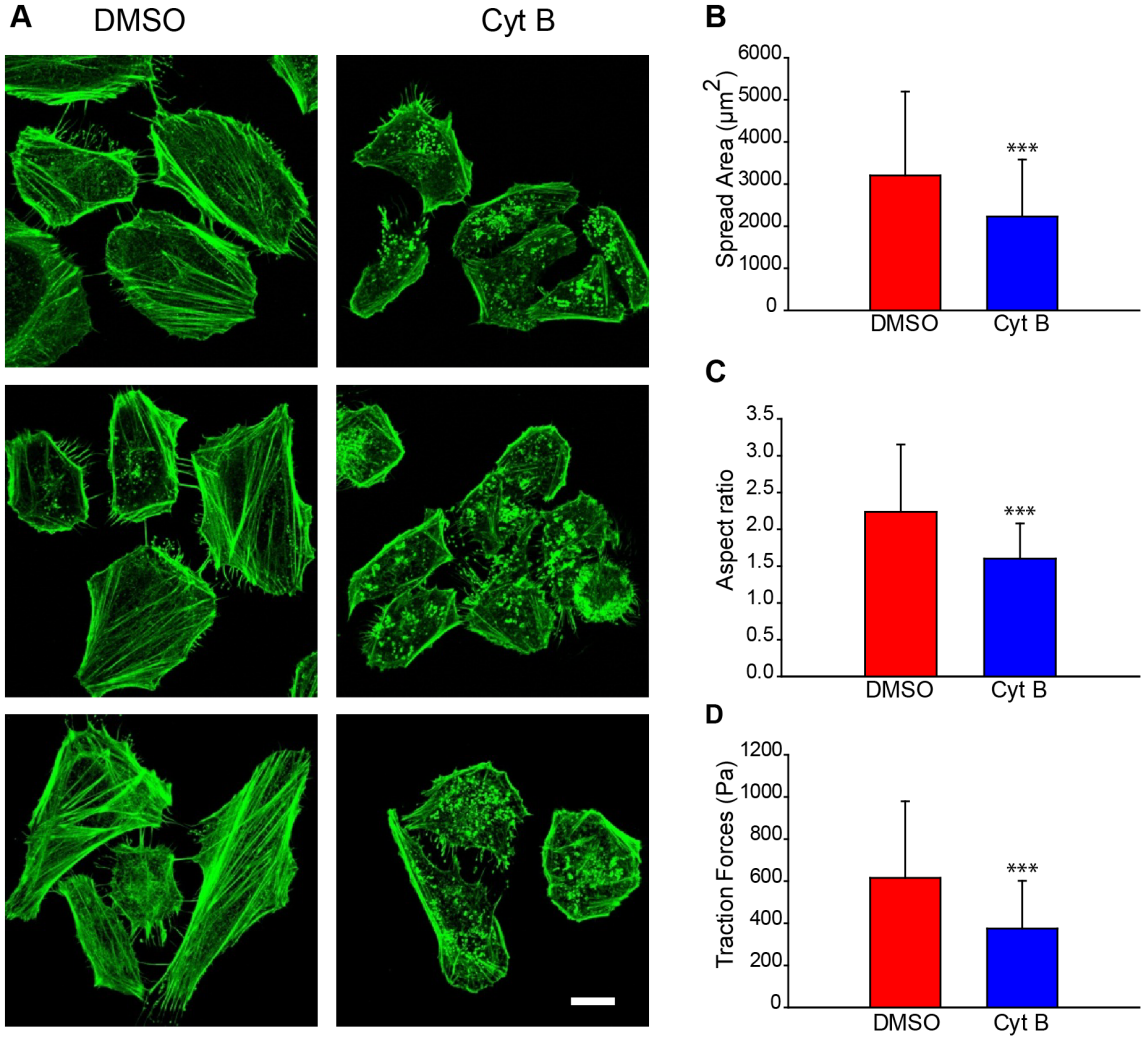
The images show that Cytochalasin B affects cellular structure and morphology and disrupts the actin cytoskeleton. Analysis of HeLa cells after 96 h of treatment. (A) Representative actin stained images of DMSO control cells (left) and 0.6 µM Cyt B-treated cells (right). B) Quantification of cellular spread area (number of cells, DMSO, n = 63; Cyt B, n = 153). (C) Quantification of cellular aspect ratio (number of cells, DMSO, n = 63; Cyt B, n = 153). (D) Cellular mean traction forces measured in 10 kPa polyacrylamide gels (number of cells, DMSO n = 79; Cyt B n = 40). Scale bar is 20 µm. ***: p<0.001.

First, the critical properties of the cellular structure and behavior were quantified for both control cells and cells treated with Cyt B, which inhibits actin polymerization and disrupts SFs [29] (**Figure 1A** **)**. Control cells were exposed to the same concentration of DMSO vehicle as Cyt B treated cells.. Control (DMSO-treated) cells exhibit a significantly higher spread area (**Figure 1B**) and are more elongated compared to the Cyt B-treated cells (**Figure 1C**). The measurement of the average cell traction forces revealed that Cyt B cells are unable to exert high forces, as shown in the literature [32] (**Figure 1D**). Therefore, Cyt B cells have a disorganized cytoskeleton that does not allow for cell spreading and the production of the same forces compared to the control cells. These results confirm that Cyt B-treated cells exhibit smaller amounts of internal stress.

### Quantification of actin stress fibers in 3D

**Figure 2:**
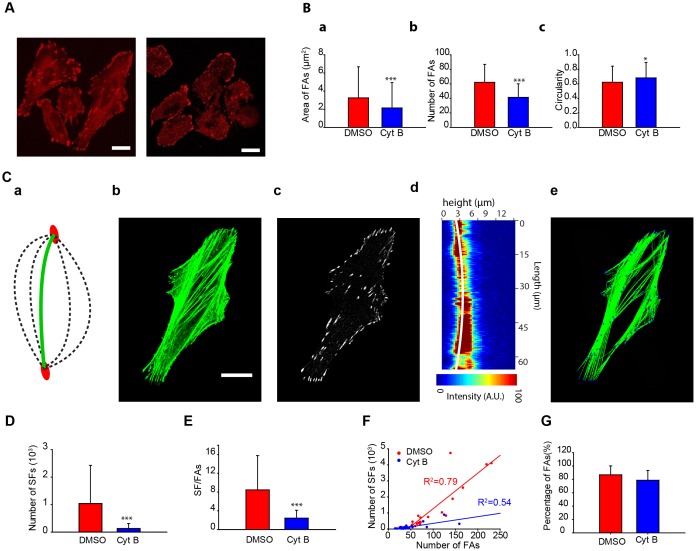
The quantification tool discerns that the effect of cytochalasin B is more pronounced on SFs than on FAs. (A) Representative images of vinculin in DMSO control cells (top) and Cyt B-treated cells (bottom). (B) Quantification of the number of FAs per cell (a), FAs area (b), and circularity (c) (number of cells: DMSO, n = 33; Cy B, n = 29). (C) (a) The stress fibers quantification tool analyses different parabolic paths (dashed arcs) between two focal adhesions (red ellipses) and selects the parabola that fulfills the intensity requirements (green arc) (Materials and methods). (b) F-actin staining and (c) vinculin staining of a cell after filtering process. (d) Representative example of a z plane containing a stress fiber and the stress fiber in 3D detected by the tool (white line). (e) Image of the stress fibers quantified by the algorithm. (D) Quantification of the number of SFs. (E) Analysis of the number of SFs depending on the number of FAs. (F) Correlation between the number of SFs and FAs. (G) Quantification of the percentage of FAs that have at least one SF (number of cells: DMSO, n = 24; Cyt B, n = 27). Scale bars are 20 µm. ***: p<0.001.

Once we confirmed that our treatment affected the actin cytoskeleton, we asked whether these changes in F-actin organization were measurable by our quantification tools. First, the cell attachment was studied. FAs are the link between actin SFs and the substrate. An image-filtering procedure was designed and applied to remove both the noise from the images taken by confocal microscopy (**Figure 2A**) and the soluble vinculin to segment the FAs. The resulting image contains all of the bright FAs and is noise free (**Figure 2C** **a**). Different properties of the FAs are quantified (**Figure 2B**) by comparing the Cyt B-treated cells and the DMSO control cells. The DMSO-treated cells have significantly more FAs per cell (**Figure 2B** **a**), and their average cell size is larger (**Figure 2B** **b**). Previous research confirmed that there are highly statistically significant differences in the number of FAs per cell when cells are under less tension [33]. However, Cyt B-treated cells have significantly more circular FAs than the DMSO control cells (**Figure 2B** **c**).

All the quantifications of SFs were done in 3D from the information obtained from confocal z-slices. To do so, a novel approach that overlaps segmented vinculin images (**Figure 2C** **c**) and actin images (**Figure 2C** **b**) was developed. FA centroids are tagged, and these positions are marked in the actin-stained images. These (X,Y) spots serve as the starting and ending points for the SFs, and the algorithm quantifies the exact number and path of each possible SF. Each possible SF is verified in the Z-axis (**Figure 2C** **d**). The expansion to 3D eliminates the 18.57±7.49% of SFs quantified in 2D. Using this software, the properties of the overlapping actin SFs were obtained (**Figure 2C** **e**). Thus, by matching possible stress fibers to focal adhesion distribution, the software estimates the number of focal adhesions present in a given region, even if their differences and overlap cannot be resolved from the images. As to the accuracy of the measurements, 6.23±3.81% of the fibers detected by the algorithm did not clearly correspond to stress fibers identified through visual inspection (**Figure S1A in File S1**). Conversely, 2.76±2.43% of visually detected fibers were not recognized by the algorithm (**Figure S1B in File S1)**. The DMSO-treated cells had about 8-fold more SFs than the Cyt B-treated cells (**Figure 2D**). Nevertheless, the fact that Cyt B-treated cells had fewer FAs could significantly influence the results. Therefore, we normalized the results by quantifying the number of SFs per each FA. In regard to the FAs, Cyt B-treated cells had 3 times fewer SFs per FA than did the DMSO-treated cells (DMSO SF/FA = 8.48±7.30; Cyt B SF/FA = 2.44±1.67) (**Figure 2E**). This normalization confirms that strong differences exist when Cyt B is added. Next, the correlation between the numbers of SFs related to FAs shows a strong positive dependency on both conditions (**Figure 2F**). When actin polymerization is inhibited, the amount of FAs decreases, and therefore there are fewer attachment points for the SFs. Additionally, most of the FAs are attached at least to one SF (DMSO 87% of FAs, Cyt B 79% of FAs) (**Figure 2G**). This result suggests that the remaining vinculin FAs may be associated with dorsal SFs [13] or that this vinculin may be related to mature focal complexes that have not yet become FAs. Taking these results together, the increase in vinculin recruitment and the number of SFs in DMSO-treated cells allow the cell to have a prominent cytoskeleton to exert higher forces and increase the cellular ability to spread [33].

**Figure 3:**
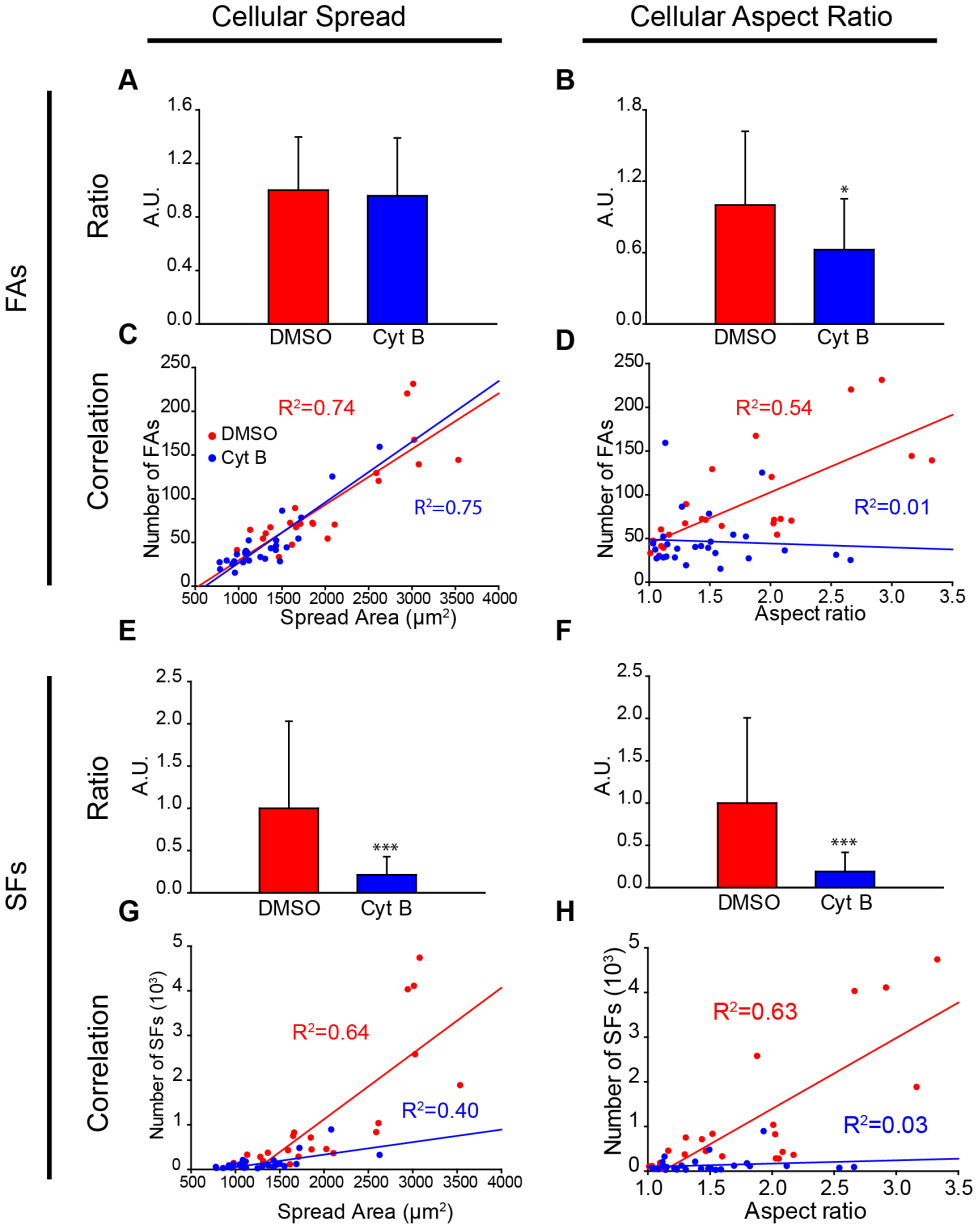
There are strong linear correlations between the number of SFs and FAs with cellular morphological features. (A) Ratio between the number of FAs per cell divided by its spread area for both DMSO and CytB condition. DMSO control cells is normalized to one. The spread area of cells similarly defines the number of FAs in both conditions. (B) Same quantification taking into account the aspect ratio of cells. Differences between both cell types are statistically significant (*: p<0.05). (C) Corresponding correlation between the number of FAs of a cell and the spread area. (D) Likewise, same correlation analysis between FAs and the aspect ratio. (E–H) Same analysis performed with SFs instead of FAs. (E–F) Ratio between the number of SFs per cell and both cellular spread area and aspect ratio reveal that DMSO-treated cells have significantly more than CytB-treated cells (***: p<0.001) (D) (number of cells: DMSO, n = 24; Cyt B, n = 27). The trend line and the coefficient of determination can be observed for each condition.

After the actin SFs and FAs were characterized, the link between these proteins and the cellular structure was quantified. Normalization and correlation were used to study the dependency of the number of SFs and FAs on the cellular spread area and aspect ratio. First, we observed that Cyt B-treated cells with the same area had the same number of FAs as the control cells (**Figure 3A**). Indeed, the number of FAs is strongly dependent on the spread area in both conditions (**Figure 3C**). Likewise, there is a strong correlation between the number of SFs and the spread area (**Figure 3G**). The control cells have a steep linear slope compared to the Cyt B-treated cells. The normalized ratio shows that the control cells have significantly more SFs, independent of the spread area (**Figure 3E**). Regarding to the aspect ratio, the control cells have more FAs and SFs than Cyt B-treated cells, independent of the aspect ratio (**Figures 3B, 3F**). The linear dependency on the number of SFs and FAs explains in the DMSO-treated cells that circular cells will have fewer cytoskeletal features. Nevertheless, this relationship cannot be extrapolated to Cyt B-treated cells because there is no correlation (**Figures 3D, 3H**). The existing balance between the properties of the cytoskeleton is affected in cells with impaired SFs.

**Figure 4:**
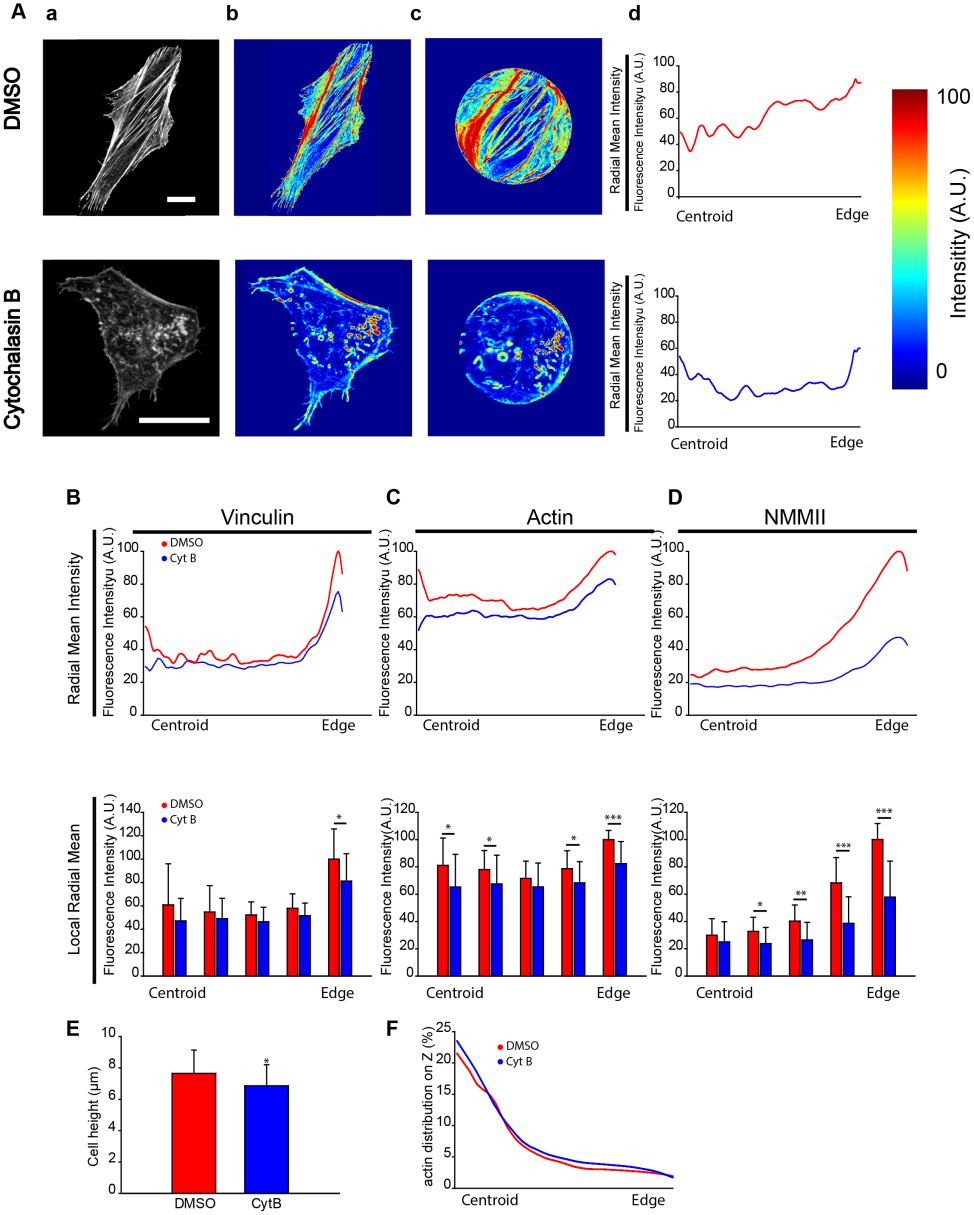
The distribution and quantification of the average intensity of critical actin SF properties reveal different protein distributions. (A) (a) Examples of measurements of the F-actin intensity of a DMSO control cell and a cell treated with Cyt B. (b) The intensity distribution of the protein in the cell (c) is normalized to a circle taking into account the distance to the centroid and the closest edge (d) and the mean along all directions is quantified obtaining the Radial Mean Intensity (RMI). (B) Top: The Radial Mean Intensity distribution of vinculin. Bottom: The Radial Mean Intensity is divided in 5 sectors to obtain the Local Radial Mean Intensity (LRMI) of vinculin. Statistical differences between both conditions were only observed in the section closest to the edge of the cell (*: p<0.05). (C–D) Same quantification for actin and Non-Muscle Myosin II. Statistical differences are observed in both proteins between Cyt-B and DMSO-treated cells (number of cells: DMSO, n = 18; Cyt B, n = 16). (E) Quantification of cell height. (F) Quantification of the percentage of total protein along the Z-axis (number of cells: DMSO, n = 29, CytB, n = 32). Scale bars are 20 µm. (*: p<0.05; **: p<0.01; ***: p<0.001).

The previous results allow justifying the differences in the SFs based on critical features such as the cell spread area or morphology. Next, we analyzed the location of critical SF proteins to gain a better insight into the cytoskeleton structure. However, a comparison of the distribution of actin, NMMII and vinculin is greatly affected by cellular shape. We thus decided to develop a new parameter (RMI, see methods) that normalizes the morphology of each cell (**Figure 4A**). This approach allows us to make comparisons among cells with both different shapes and different dimensions to obtain systematic and high-throughput results. This method can be applied to study any type of structure or protein within a cell. In this study, we examined the distribution of actin SFs and vinculin. Previous studies have identified a close relationship between the force applied by a SF and FA localization [19] as well as between NMMII distribution and spacing [21].

With regard to vinculin, we observed that there are strong differences in the RMI between the Cyt B and DMSO-treated cells near the nucleus and close to the edge (**Figure 4B** **)**. Measurements of the gradient confirm these differences (slope, DMSO 72.11°, Cyt B 62.50°) (**Figure S2 in File S1**). When the cell studied is divided into 5 sections (using the LRMI parameter, see methods), significant differences were found in the area closer to the edge. Moreover, if the total intensity of vinculin is considered and analyzed per section (using the CARIP parameter, see methods), we also detected statistically significant differences near the nucleus (**Figure S3A in File S1**). Indeed, 31% of the protein in control cells versus 23% of the protein in Cyt B-treated cells is present in this area. As far as actin is concerned, there are meaningful differences in CARIP (**Figure S3B in File S1**) next to the edge and on the second section (closer to the centroid). This difference likely occurs because when the actin cytoskeleton is inhibited, the F-actin distribution is homogenized across the cell. In addition, when the LRMI was analyzed, significant differences were also observed in the area close to the nucleus and the edge. The RMI confirmed the trend with similar differences between the two conditions, with exception of the increase in mean intensity near the centroid and the edge in the DMSO-treated control cells (**Figure 4C**). Measurement of the gradient confirms that the constant differences are maintained between both conditions (slope, DMSO 46.39°, Cyt B 37, 38°) (**Figure S2 in File S1**). Then, NMMII RMI was analyzed to gain a better insight into SF distribution (**Figure 4D**). Interestingly, there is significant recruitment of NMMII in the area near the edge of cells (**Figure S4 in File S1**), whereas the intensity of NMMII decreases meaningfully when moving away from the periphery, as expected [19]. Indeed, the intensity is minor and remains stable from half the cell to the centroid. Conversely, cells treated with Cyt B do not significantly recruit NMMII near the periphery of the cell (slope, DMSO 52.25°, Cyt B 37.86°) (**Figure S2 in File S1**). LRMI highlights the highly significant differences in the NMMII distribution. However, when normalized to the total intensity, the variances in CARIP are minor across the cell (**Figure S3C in File S1**). Therefore, there are major differences in the LRMI of actin and NMMII across the cell. Conversely, FAs are mainly in the periphery and close to the centroid of cells, similar to F-actin.

These results show that SFs tend to start on the peripheral region of the cell and end either on the periphery or near the nucleus. Furthermore, the NMMII distribution of Cyt B-treated cells compared to DMSO-treated cells highlights significant differences close to the periphery. This result suggests that in cells that are supporting a higher tension with a prominent cytoskeleton, NMMII locates closer to the peripheral region of the cell. This finding confirms those of previous research [21], [34]–[36]. Our study concluded that the inhibition of actin polymerization hinders NMMII recruitment near the edge, which is confirmed by the drop in the cellular ability to exert force (**Figure 1D**).

We expanded this method to study the distribution of actin with the height of the cell (Supplementary materials and methods). There are statistical significant differences in the height of cells (**Figure 4E**). We normalized the height of the cell to study the actin distribution. There were no differences in the actin distribution with the height of cells between DMSO-treated and CytB-treated cells, when the percentage of protein was studied (**Figure 4F**).

## Discussion

Our work presents a unique image-based computational tool to evaluate the role of FAs and SFs. This tool may also be applied to quantify and analyze the distribution of different cell proteins within the actin cytoskeleton, microtubules or intermediate filaments.

First, we quantified and measured the distribution of actin SFs in 3D, combining both FA and acto-myosin images, which offers a new reliable approach to study the actin organization. To do so, our methodology combines quantification with the normalization of the cell shape to correlate both data sets. We tried to establish a clear relationship between cellular morphology (both aspect ratio and spread area) with the number and distribution of SFs where a certain correlation was observed. As indicated, Cyt B was added to the cells to compare the structural trends with respect to the control cells. This drug leads to the disorganization of the cytoskeleton and to the significant formation of actin-based aggregates that the software clearly distinguishes. In this case, the software is notably useful as the algorithm discriminates between fibers and large intense aggregates regardless of intensity levels.

Different methodologies have been previously followed to study cytoskeletal features. For example, the spacing along the SFs of critical proteins such as α-actinin and myosin has been widely studied [19], [21]. Another frequently used analysis involves actin-stained image processing using high thresholds to eliminate lower values of actin intensity [19]. Such filtering has been applied to study single cell orientation and SF direction after cyclic stretch [37]–[40], or on a patterned substrate [41]. Alternatively, Prager-Khoutorsky and co-workers measured the percentage of cells with long stress fibers to avoid the difficulty generated by overlapping fibers [17]. As to distribution, distance analyses of the 3D nuclear radial distribution of fluorescence intensity have been used to study telomeres and their clusters [42]. Recent research by Möhl et al. [23] normalized the shape of migrating keratinocytes to a circle to be able to link data from separate experiments and techniques, and to study protein distribution with respect to the direction of migration. Likewise, the fluorescence emission of solid lipid nanoparticles was studied by normalizing one fluorescence intensity line per cell [24]. In comparison to this previous work, our approach introduces useful and novel elements. First, we designed a novel automated analytical tool to quantify SFs in 3D, which does not lose any information through image filtering and which solves the issue of overlapping SFs by linking vinculin and actin images. Linking both images allows us to provide local values of each SF in three dimensions. Importantly, the quantification is automated and controlled through a few parameters, thereby avoiding subjectivity. Second, this quantification is combined with normalized distribution analyses, which can be applied to any cellular structure in 3D. Even though we did not focus on migrating cells, these tools could be easily applied to cells in movement by taking a migration direction as reference (as done by Möhl et al.) or by dividing cells in different angular sectors (**Figure S5 in File S1**).

In summary, we quantified the critical features of the actin SFs. Previous studies include few quantitative results relating actin SFs and the cellular structure. Indeed, the main results are related to FAs. However, our methodology allows us to study the relationship among the number of SFs, FAs and critical cell morphological features, such as the aspect ratio and the spread area. For instance, we observed that the spread area highly correlates with the number of focal adhesions independently of the treatment. In contrast, correlations between SFs/FAs and cell aspect ratio only appeared in non-treated cells. Since cell aspect ratio and stress fiber formation have been linked to functions such as cell polarization and mechanosensing [17]–[43] or invasive capacity [44]–[45], the correlative information provided by our tools could potentially be useful to address relevant biological questions. Our analysis of the local intensity distribution provides key complementary information to better interpret the results. Furthermore, the quantification of the distribution can be used for cells, proteins or structures, independent of their morphology. Furthermore, both static and dynamic studies can be conducted using this method.

## Conclusions

In conclusion, we have developed two robust computational tools that work in a complementary fashion, allowing for integral data analysis encompassing the main quantitative features of normalization, correlation and distribution. The versatility of the tools permits a broad range of applications. The quantification of SFs is, to our knowledge, the first computational tool that automatically measures the properties of SFs from raw images, correlating them with cell features. We propose that our methodology, which takes advantage of both focal adhesion and actin staining, can be used directly within a broad range of experiments to study the importance of ventral SFs. Conversely, the significant differences observed in the location of different critical proteins highlight the importance of structure distribution analysis as a meaningful tool. Indeed, we believe that real-time experiments where the movement of the structures could be tracked may make the most of this distribution tool through normalization. This work highlights the importance of the extensive characterization of the condition being studied. We suggest that the synergy of the standardization results in a powerful approach to compare numerous and major cell features.

## Supporting Information

File S1 **Supporting figures.** Figure S1, Examples of misidentified fibers. (A) Left, actin stained image. Center and right, actin images superimposed with examples of fibers that fulfill the requirements established but are incorrectly taken into account as SFs by the algorithm (in green). The fiber in the center actually corresponds to two separate fibers, and the one in the right has a zone with no underlying staining. (B) Example of SFs not detected by the algorithm. Left, actin stained image and right, SFs result obtained by the algorithm. The SFs observed in the actin stained image (center) inside the rectangle are not detected by the algorithm (right). Scale bars are 20 µm. Figure S2, The radial Mean Intensity at the cell edge is fitted to a second order polynomial of the form ax^2^+bx+c where the starting point is automatically selected. (A) Results of the starting point, where intensity starts to increase significantly, polynomial coefficients and average slope for vinculin, actin and NMMII. (A) The Radial Mean Intensity data at the cell edge fitted for vinculin, actin and NMMII for DMSO-treated cells. (B) Same fitting for CytB treated cells. A comparison between both conditions is shown in (D). Figure S3, Percentage of total intensity distribution across the cell shows significant differences in the edge of the cell. (A) Quantification of the percentage of vinculin located in sections with the same area for both DMSO and CytB-treated cells. DMSO-treated cells have a significant increase in the Constant Area Ring Intensity Percentage (CARIP) localization of vinculin in the edge compared to Cyt B-treated cells (number of cells: DMSO, n = 26; Cyt B, n = 23). B) Likewise, the sector with the highest amount of actin intensity is the edge (number of cells: DMSO, n = 25; Cyt B, n = 25). (C) There are no significant differences in the localization of NMMII between DMSO control cells and CytB-treated cells (number of cells: DMSO, n = 18; Cyt B, n = 16). Figure S4, There is a significant increase in the amount of NMMII closer to the edge of the cell. Representative images of NMMII and vinculin of DMSO- and CytB-treated cells. Scale bar is 20 µm. Figure S5, Measurement of angle dependent actin distribution. (A) Example image of DMSO-treated cell divided into four different sectors: two in the direction of the principal axis of the cell, and another two perpendicular to it (B) Intensity distribution of the cell. (C) Cell shape normalized to a circle. (D) Colormap of the Radial Mean Intensity quantified for the four different sectors. (E) Colormap of the Radial Mean Intensity in the cell with the original shape for the four different sector. (F) Measurement of the Radial Mean Intensity for each sector.
(PDF)

File S2 **Supplementary Material and Methods.**
(DOCX)
