# Supplementary material for: Image Analysis for the Quantitative Comparison of Stress Fibers and Focal Adhesions
Source: PLoS One. 2014 Sep 30;9(9):e107393. doi: 10.1371/journal.pone.0107393 (PMC4182299; doi:10.1371/journal.pone.0107393)
Supplement: File S1 — Supporting figures. Figure S1, Examples of misidentified fibers. (A) Left, actin stained image. Center and right, actin images superimposed with examples of fibers that fulfill the requirements established but are incorrectly taken into account as SFs by the algorithm (in green). The fiber in the center actually corresponds to two separate fibers, and the one in the right has a zone with no underlying staining. (B) Example of SFs not detected by the algorithm. Left, actin stained image and right, SFs result obtained by the algorithm. The SFs observed in the actin stained image (center) inside the rectangle are not detected by the algorithm (right). Scale bars are 20 µm. Figure S2, The radial Mean Intensity at the cell edge is fitted to a second order polynomial of the form ax2+bx+c where the starting point is automatically selected. (A) Results of the starting point, where intensity starts to increase significantly, polynomial coefficients and average slope for vinculin, actin and NMMII. (A) The Radial Mean Intensity data at the cell edge fitted for vinculin, actin and NMMII for DMSO-treated cells. (B) Same fitting for CytB treated cells. A comparison between both conditions is shown in (D). Figure S3, Percentage of total intensity distribution across the cell shows significant differences in the edge of the cell. (A) Quantification of the percentage of vinculin located in sections with the same area for both DMSO and CytB-treated cells. DMSO-treated cells have a significant increase in the Constant Area Ring Intensity Percentage (CARIP) localization of vinculin in the edge compared to Cyt B-treated cells (number of cells: DMSO, n = 26; Cyt B, n = 23). B) Likewise, the sector with the highest amount of actin intensity is the edge (number of cells: DMSO, n = 25; Cyt B, n = 25). (C) There are no significant differences in the localization of NMMII between DMSO control cells and CytB-treated cells (number of cells: DMSO, n = 18; Cyt B, n = 16). Figure S4, There is a si [file pone.0107393.s001.pdf]

Fig.S1

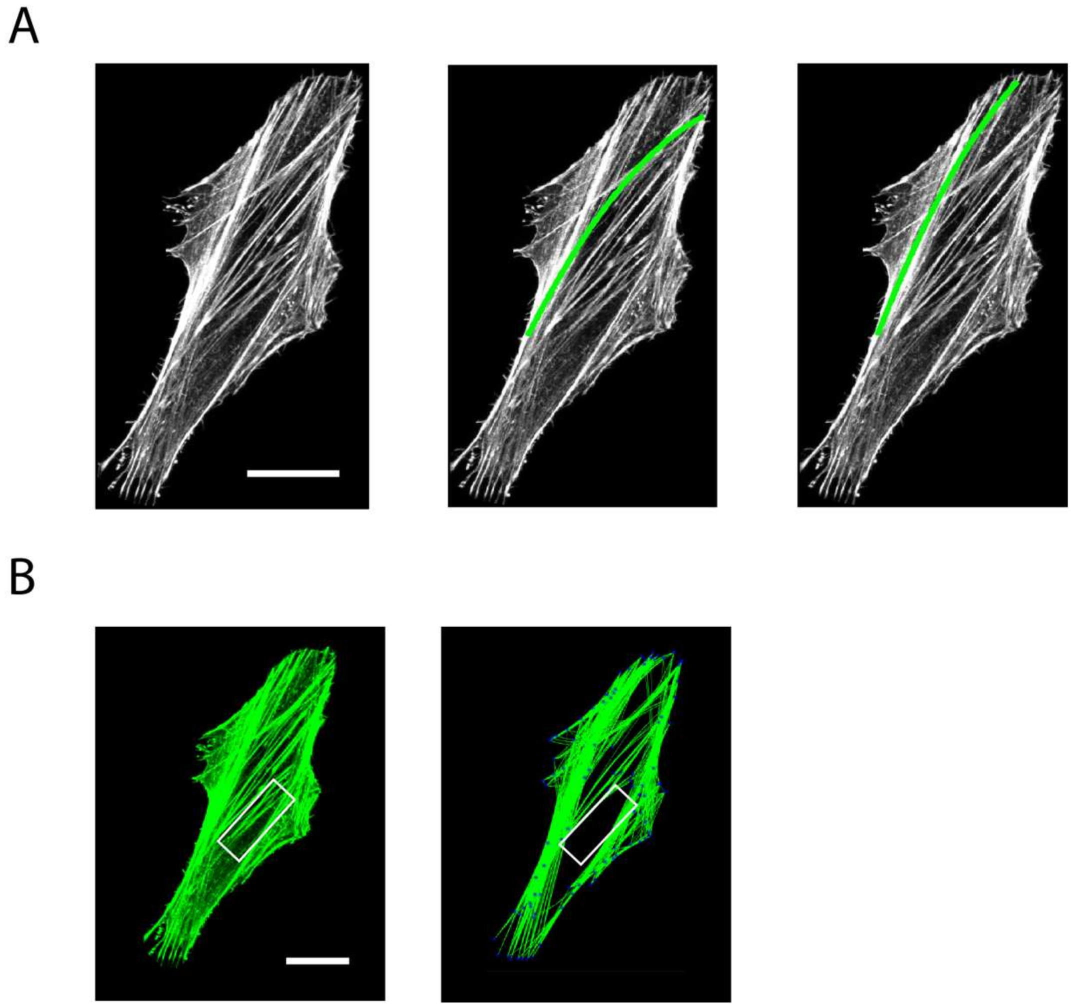

Fig.S2

A

Vinculin

	DMSO	Cyt B
Start Point	730	700
Polynomial (a,b,c)	(0.1597, -1.2863, 38.1771)	(0.0819, -0.6073, 32.6737)
Average Slope	72.11°	62.50°

Actin

	DMSO	Cyt B
Start Point	520	570
Polynomial (a,b,c)	(0.0243, -0.2550, 64.8464)	(0.0216, -0.4331, 61.0742)
Average Slope	43.93°	37.38°

NMMII

	DMSO	Cyt B
Start Point	320	520
Polynomial (a,b,c)	(0.0222,-0.4147, 29.5144)	(0.0194,-0.2155, 20.1088)
Average Slope	52.25°	37.86°

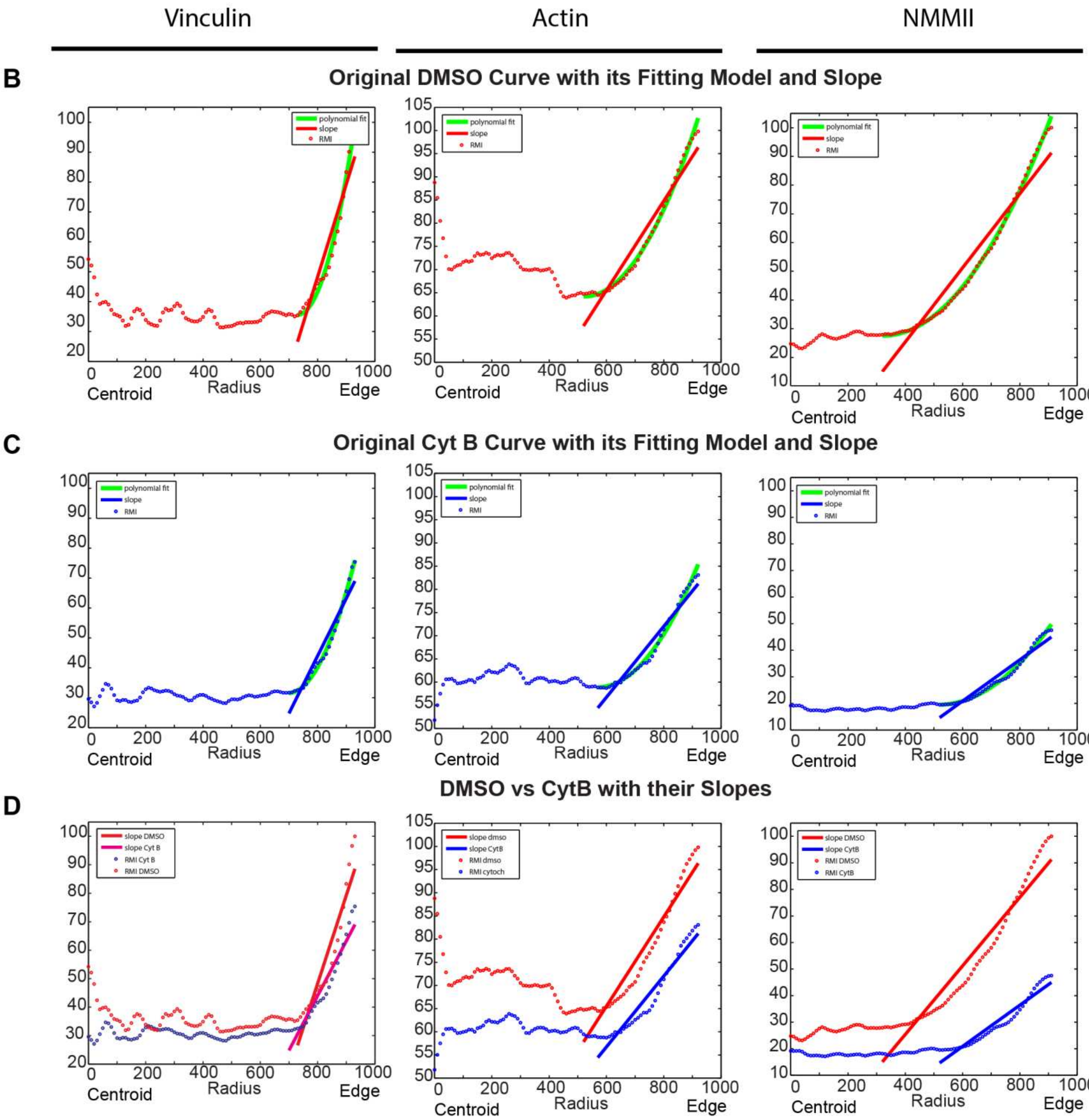

Fig.S3

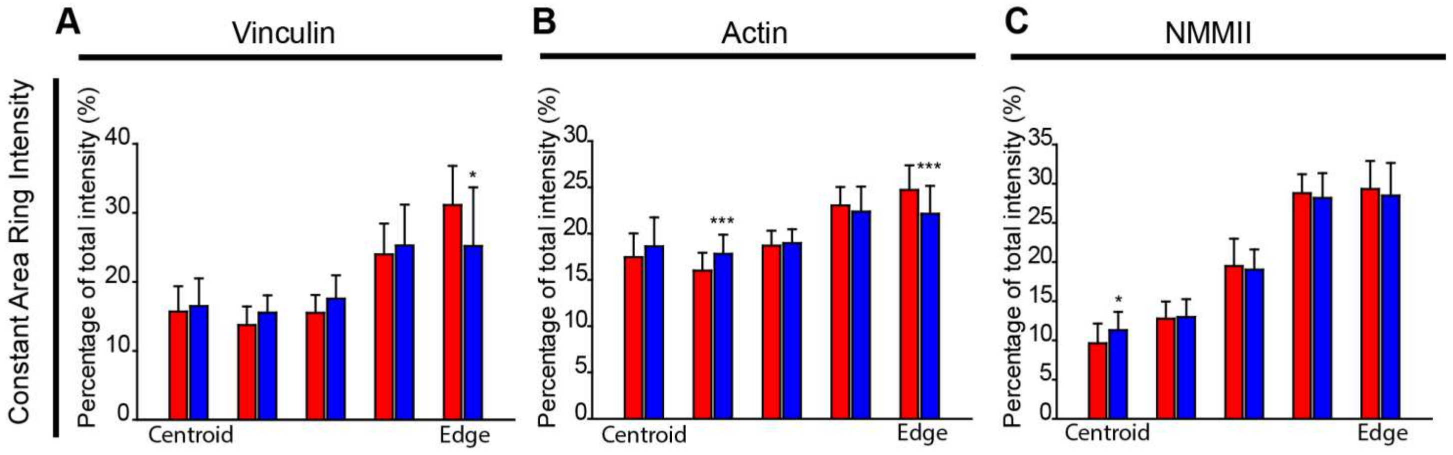

A
Vinculin
B
Actin
C
NMMII
Constant Area Ring Intensity
Percentage of total intensity (%)
0
10
20
30
40
Centroid
Edge
*
0
5
10
15
20
25
30
***
***
0
5
10
15
20
25
30
35
*

Fig.S4

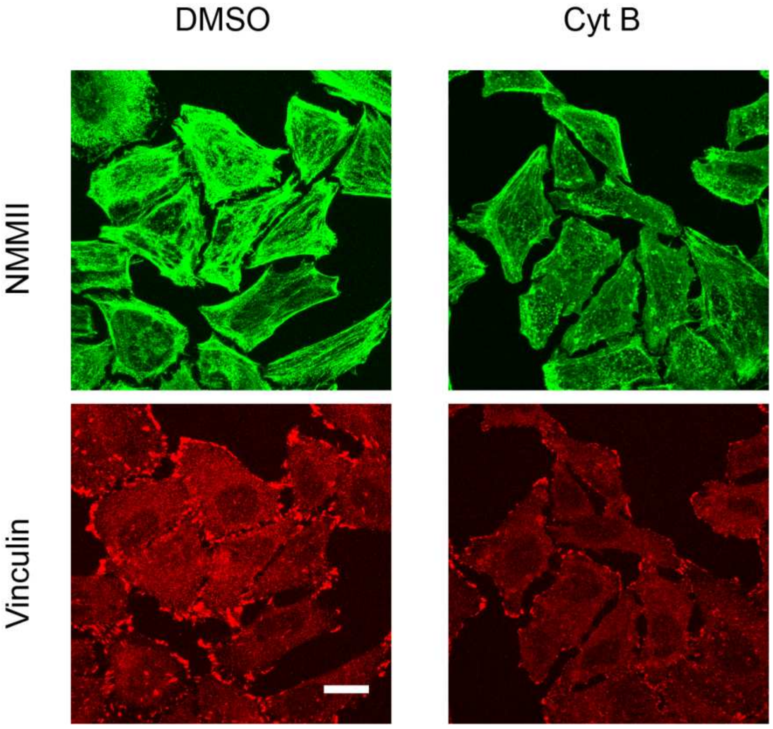

Fig.S5

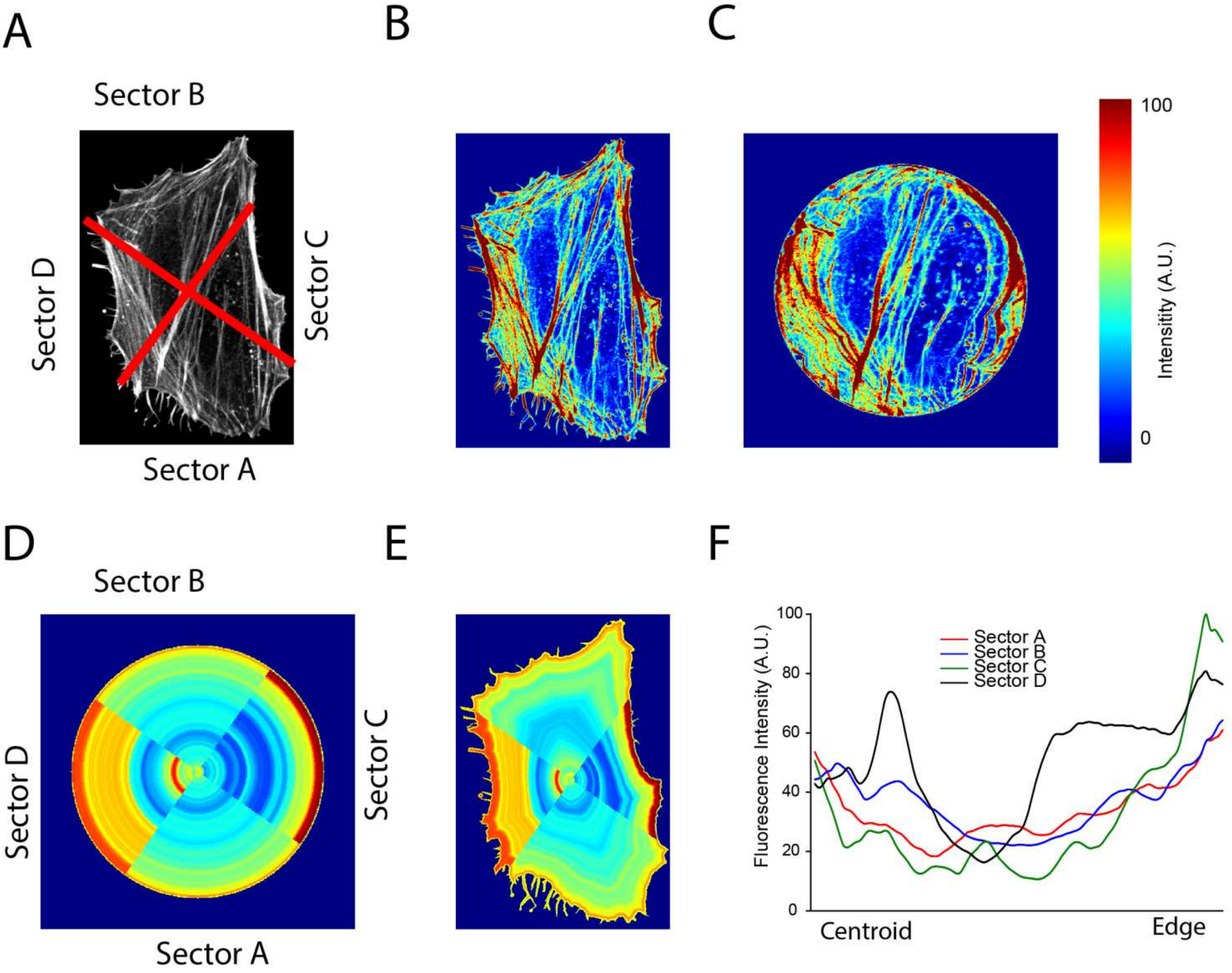
A
B
C
Sector B
Sector D
Sector C
Sector A
100
Intensity (A.U.)
0
D
E
F
Sector B
Sector D
Sector C
Sector A
Fluorescence Intensity (A.U.)
100
80
60
40
20
0
Sector A
Sector B
Sector C
Sector D
Centroid
Edge
