# Supplementary material for: Image Analysis for the Quantitative Comparison of Stress Fibers and Focal Adhesions
Source: PLoS One. 2014 Sep 30;9(9):e107393. doi: 10.1371/journal.pone.0107393 (PMC4182299; doi:10.1371/journal.pone.0107393)
Supplement: File S2 — Supplementary Material and Methods. (DOCX) [file pone.0107393.s002.docx]

**SUPPLEMENTARY MATERIALS AND METHODS**

**Traction Force Microscopy**

Polyacrylamide gels were prepared as previously described (1). Briefly, glass bottom Petri dishes (MatTek) were treated with 0,1N NaOH (Sigma-Aldrich) for 20 min, 5 min with 3-aminopropyltrimethoxysilane (Sigma-Aldrich) and 30 min with 4% (wt/vol) glutaraldehyde (Panreac). Then washed with 70% (vol/vol) ethanol, sonicated for 5 min, and air-dried.

The 10-kPa gels were prepared mixing ultrapure water, Acrylamide solution (Bio-Rad) and N-N′-methylene-bis-acrylamide (Bio-Rad), tetramethylethylenediamine (1/2000; Bio-Rad) and free radical ammonium persulfate (10% solution, 1/100 vol/vol; Bio-Rad) were added for gel polymerization and the solution was filtered (0.22-μm filter) before adding fluorescent polystyrene microbeads (1.1-μm diameter) (Invitrogen) diluted 1:100. Small droplets of the solution were dispensed onto the treated glass-bottomed Petri dishes, flattened using a 12 mm diameter cover-slip, and incubated at room temperature for 15 min for gel polymerization. Then, top coverslips were removed. Gels were activated with 0.5 mg/mL sulfo-SANPAH (Fisher) and introduced in the UV chamber for 4 min. After washing gels, three times with Hepes (50 mM), the gel was coated with fibronectin (1.77 μg/cm^2^; Millipore) for 1 h at 37ºC. Finaly, gels were rinsed three times with Hepes and one with PBS before addition of the culture medium.

The macroscopic elastic shear modulus of each gel was measured as described previously [1]. Briefly, an Anton Paar Physica MCR 301 rheometer with 25-mm parallel plate geometry was used to measure rigidity of gels at 37 ºC. Amplitude sweeps were used to identify the linear regime; frequency sweeps at 5% were then used to extract storage, loss, and complex moduli of each sample. At least three independent samples were measured per condition.

5,000 cells/cm2 were seeded on polyacrylamide gels at a density of 12 h before performing the measurements. At least 20 fields of interest per sample were studied using a 40× objective in a Nikon Ti-Eclipse microscope with simultaneous phase and epifluorescence illumination. Cells were afterwards treated with 0.5% SDS detergent and a second epifluorescence image was taken. After registering the first and second images, the displacement of each bead relative to its undisturbed position was computed using a program based in the Butler method [2]. The results quantified are whole-cell traction force average stress measurements, assuming that all of the tractions outside the area of the cell must be zero.

**Z Intensity Distribution**

To study the percentage of total protein distribution along cell height, we calculate the Z intensity percentage (ZIP) as:

where I_3D_ is the z-stack acquired between the bottom and the top of the cell under study.

Note that ZIP depends on the total number of Z-planes used to image the cell, which, in turn, varies with both the cell height and the Z-sampling used during the image acquisition. Therefore, to allow comparative studies among different cells, we first normalize the cell height and then interpolate to calculate ZIP at equally spaced height positions.

**Analysis of the Radial Mean Intensity profile**

To analyze the Radial Mean Intensity (RMI) profile at cell edge, we fit it to a second order polynomial of the form $p=ax^{2}+bx+c$. The starting point at which the RMI data is considered for the fitting can be automatically calculated as the point that minimizes the error between p and the RMI profile at cell edge. Finally, the average slope of the fitted region is obtained by performing a second fit to a first order polynomial.

**Direction-dependent local intensity quantification**

Local intensity quantification tool has been developed to analyze and compare cells of different sizes and morphologies independently of their orientation or mechanical cycle. Nevertheless, it can be also used to quantify the labeled cell structure in any given direction of interest, such as the principal axis of cell or the migration direction. To do so, the circular intensity distribution of the mapped cell image is divided into different angular sectors that are independently analyzed. Typically, four sectors of 90° are considered: two aligned with the direction of interest and another two perpendicular to it (see Figure S4 in File S1).

**References**

**1.-** Van Damme P, Lasa M, Polevoda B, Gazquez C, Elosegui-Artola A, et al.(2012). N-terminal acetylome analyses and functional insights of the N-terminal acetyltransferase NatB. Proc Natl Acad Sci U S A. 109: 12449-54

**2.-** Butler JP, Tolić-Nørrelykke IM, Fabry B, Fredberg JJ (2002). Traction fields, moments, and strain energy that cells exert on their surroundings. Am.J.Physiol.Cell Physiol. 282: C595-C605.
